# Antigenicity Alternations of Variant PEDV S Protein Disclosed by Linear B Cell Epitope Mapping

**DOI:** 10.3390/v14071371

**Published:** 2022-06-23

**Authors:** Ruisong Yu, Shijuan Dong, Bingqing Chen, Yingjie Liu, Fengping Li, Fusheng Si, Chunfang Xie, Zhen Li

**Affiliations:** Institute of Animal Husbandry and Veterinary Science, Shanghai Key Laboratory of Agricultural Genetics and Breeding, Shanghai Engineering Research Center of Breeding Pig, Shanghai Academy of Agricultural Sciences (SAAS), Shanghai 201106, China; yuruisong@saas.sh.cn (R.Y.); dsjuan@saas.sh.cn (S.D.); chenbingqing@saas.sh.cn (B.C.); 18769763823@163.com (Y.L.); 18354226811@163.com (F.L.); mr.fusheng@saas.sh.cn (F.S.); xiechunfang@saas.sh.cn (C.X.)

**Keywords:** porcine epidemic diarrhea virus, spike protein, epitope mapping, antigenic alteration

## Abstract

The spike protein (S) plays a crucial role in porcine epidemic diarrhea virus (PEDV) infection and induces neutralizing antibodies. Mutations of the S protein are supposed to provide the main antigenic shift leading to the antigenic escape of PEDVs. It is therefore a significant question how much accumulation of antigenic shift could lead to the antigenic escape of the variant PEDV. To provide an answer in the study, B cell epitopes (BCEs) on the S protein of the PEDV vaccine strain CV777 (S^CV777^) and variant strain SD2014 (S^SD2014^) were mapped using biosynthetic peptides and rabbit anti-PEDV S serum. Seventy-nine and 68 linear BCEs were identified from S^CV777^ and S^SD2014^, respectively. While 66.2% of the BCEs of S^SD2014^ could be recognized by anti-S^CV777^ serum and 67.1% of S^CV777^ BCEs could be recognized by anti-S^SD2014^ serum, more than 40% of the BCEs identified using anti-S^CV777^ serum on S^CV777^ could not be recognized by anti-S^SD2014^ serum and vice versa. The completely shared BCEs took low percentages of 29.4% and 25.3% for S^SD2014^ and S^CV777^, respectively. These results indicate a low conservation of antigenicity of the S protein compared to a relatively high amino acid sequence similarity of 92.2% between the two strains. The study provided a BCE shift reference of PEDV antigenic escape and surveillance control.

## 1. Introduction

Porcine epidemic diarrhea virus (PEDV), a member of *Alphacoronavirus* in the family Coronaviridae, causes acute and highly contagious enteric disease in pigs and is designated as porcine epidemic diarrhea (PED). The pathogen was first detected in Europe in the early 1970s [1,2]. Since the 1980s, PED has become a severe problem in pig-producing countries in Europe and Asia, especially in Asia, including China, Korea, Japan, and Thailand [1,3]. In late 2010, a novel variant of PEDV emerged in Asia and became prevalent in swine herds worldwide [2,4,5]. Variants of PEDV caused failure of vaccination, which employed traditional vaccine technology and classical PEDV strains and brought great economic loss to the livestock industry.

Among the structural proteins of PEDV, the spike (S) protein plays pivotal roles in PEDV attachment, receptor binding, and virus–cell membrane fusion during PEDV invasion into host cells [6]. The PEDV S protein is also involved in the induction of neutralizing antibodies in the host [7], so for this reason the S protein also represents one of the most genetically unstable structural proteins of PEDV. In addition to the typical amino acid (aa) “deletion and insertion”, many aa substitutions are found scattered across the S protein of field strains as compared to the classical strain CV777 [3,8]. Given the important role in the induction of neutralizing antibodies, there is a high probability that the variations of the S protein contribute to the main antigenicity shift and antigenic escape of the PEDV field strain.

Important neutralizing regions had been screened out from the PEDV S protein, such as the N-terminal domain [9,10], CO-26K equivalent (COE) epitope [7,9,10], S1D epitope [11,12], and C-terminal epitope [13,14]. These regions, however, also carry the most mutations in circulating PEDVs due to high immune pressure [15,16,17,18]. The comparison of antigenicity contour and composition between vaccine and wild-type PEDV was important for understanding the emergence of virulent strains and the mechanisms of unsuccessful vaccination. Such studies would also be of great importance for vaccine development and antibody detection.

The highly virulent PEDV SD2014 strain was the etiology of a PED epidemic in a farm in Shandong Province of China in 2014. Whole-genome phylogenetic analysis showed that PEDV SD2014 belongs to the Genotype II subclass, clustered together with the re-emergent virulent variant PEDV strains, including US strains. Compared to the S protein of the classical strain (Genotype I), the S protein of SD2014 strain (S^SD2014^) has the typical insertion and deletion found in the S protein of current epidemic strains. In the present study, fragments of S proteins of the PEDV classical vaccine strains CV777 (S^CV777^) and S^SD2014^ were expressed and purified to immunize rabbits. Using the polyclonal sera acquired, the linear B cell epitopes (BCEs) were identified from serial glutathione S-transferase (GST)-fusion expressed 16-mers covering the entire sequences of S^CV777^ and S^SD2014^. Recognition efficiency of the anti-sera to the BCEs was analyzed on the two S proteins. The results confirm the low conservation of antigenicity of the S protein of the wild-type PEDV strain SD2014 compared with the classical strain CV777. The study provided a clear BCE comparison of the S proteins of the two PEDV strains, which laid a solid foundation for antigenicity variation and an antigenic escape analysis of wild-type PEDV.

## 2. Materials and Methods

### 2.1. B Cell Epitope Prediction

Sequence alignment of S^CV777^ (ALS35469.1) and S^SD2014^ (AND76936) was performed with Mega 7 (Temple University, Philadelphia, PA, USA). Online server IEDB (Immune Epitope Database and Analysis Resource, http://www.iedb.org/home_v3.php (accessed on 17 January 2022)) was used for BCE prediction. Seven residue-length epitopes were viewed as adequate to persuade defensive immune reaction. Only those epitopes predicted by no less than three algorithms were chosen. VaxiJen 2.0 server (http://www.ddg-pharmfac.net/vaxijen/VaxiJen/VaxiJen.html (accessed on 24 January 2022)) was utilized for antigenicity analysis of chosen epitopes.

### 2.2. Preparation of Recombinant PEDV S Protein

Recombinant S protein (S^CV777^ or S^SD2014^) employed as the immunization antigen was prepared as described previously [19]. Briefly, the codon-optimized *S* gene was synthesized, cloned into pET-28a or pET-32a in three segments (*sf1*, 1 to 1488 nt; sf2, 1441 to 2928 nt; and *sf3*, 2881 nt to the end), and expressed in *Escherichia coli* (*E. coli*) BL21. After separation of the expressed protein by SDS-PAGE and purification by electro-elution of the excised gel band, the protein segment was concentrated with PEG 8000 and stored at −80 °C prior to use.

### 2.3. Immunization of Animals

Nineteen female New Zealand white rabbits (~2 kg body weight) were purchased from the Shanghai SLAC Laboratory Animal Co. Ltd. (Shanghai, China). The trial was approved by the Institutional Animal Care and Use Committee of Shanghai Academy of Agricultural Sciences (SAAS, Shanghai, China) (protocol code: SAASPZ0521023, 21 February 2021), and animals were treated in accordance with the regulations and guidelines of the committee. Eighteen rabbits were used for the immunization with the six S protein fragments (SFs) prepared as in 2.2 (3 fragments per S protein, SF1 to SF3 and 3 rabbits for each SF). Three rabbits were injected intramuscularly with 0.5 mg of each purified recombinant SF (SF1, SF2, or SF3) emulsified in complete Freund’s adjuvant (CFA) (Sigma_Aldrich, San Francisco, CA, USA) at multiple sites on the back of each animal. Three booster injections of 0.25 mg of the same antigen per injection in incomplete Freund’s adjuvant were administered at two-week intervals. Animals were bled 7 days after the third booster, and the serum was separated and stored at −80 °C until use. The mixed sera from 3 rabbits immunized with each SF were used in the following-up tests. Non-immune serum from the remaining one rabbit that received only CFA was used as the negative control.

### 2.4. Biosynthesis of 16-Mer Peptides

The truncated glutathione S-transferase (the initial 188 aa, GST188) was used as carrier for the expression of 16-mer peptides. One hundred and seventy-one 16-mer peptides and one 14-mer peptide with an overlap of 8 aa residues covering the entire S^CV777^ (or 172 16-mers and one 10-mer for S^SD2014^) were expressed as fusion protein as described earlier [19]. Briefly, the synthesized and then annealed DNA fragments encoding each a 16-mer or 14-mer or 10-mer peptide, incorporated with *Bam*H I and TAA-*Sal* I cohesive ends on their 5′- and 3′-terminals, respectively, were inserted into the *Bam*H I and *Sal* I sites of plasmid pXXGST-1. The confirmed recombinant plasmids were transformed into *E. coli* BL21 and temperature-induced to express the fusion peptides. Positive clones were selected by SDS-PAGE. Cell pellets containing each fusion peptide were stored at −20 °C until BCE mapping by Western blot (WB).

### 2.5. SDS-PAGE and WB

Cell pellets were resolved by SDS-PAGE using 15% gels. Gels were either stained with Coomassie Brilliant Blue G-250 (Sigma_Aldrich, San Francisco, CA, USA) for confirming the bands of fusion proteins or processed for WB by electro-transferring the proteins onto 0.2 μm polyvinylidene difluoride (PVDF) membrane (Immobilon^®^-P^SQ^, Merck Millipore Ltd., Darmstadt, Germany). Membranes were then blocked with 5% (*w*/*v*) skimmed milk powder in tris-buffered saline with 0.1% tween 20 (TBST), incubated sequentially with anti-S serum (dilution, 1:4000 in TBST) and goat anti-rabbit IgG conjugated to horseradish peroxidase (dilution, 1:80,000 in TBST) (Protein Tech Group, Inc., Chicago, IL, USA), and visualized by enhanced chemiluminescence using ECL plus Western blot detection reagent (Pierce^TM^, Rockford, IL, USA) according to the manufacturer’s instructions.

### 2.6. Immunofluorescence Assay (IFA)

Vero cells were seeded in a 24-well plate and infected with attenuated PEDV DR13 (belonging to the GI subclass, kept in our laboratory) when the cells reached approximately 80% confluence. At 18 h post infection, the cells were fixed with 4% paraformaldehyde for 15 min and then permeabilized with 0.1% Triton X-100 for 15 min at room temperature. After being washed three times in phosphate-buffered saline (PBS), the cells were blocked with 5% goat serum in PBS for 1 h at 37 °C and then incubated with rabbit anti-SFs serum (harvested in Section 2.3; dilution, 1:2000 in 5% goat serum) for 50 min. After three washes with PBS, cells were incubated with Alexa Fluor 647 goat anti-rabbit IgG (H + L) antibody (Beyotime Biotech Inc., Shanghai, China) in the dark for 45 min at 37 °C. Following several washes, cells were stained with 4′, 6-diamidion-2-phenylindole (DAPI, Merck, Darmstadt, Germany), and the fluorescence was visualized with Zeiss Axio Scope A1 microscope (Carl Zeiss, Oberkochen, Germany).

### 2.7. Immunohistochemistry Assay (IHC)

For IHC, Vero cells grown in 24-well plate were infected with attenuated PEDV DR13. At 18 h post infection, the cells were fixed and permeabilized and then air dried and incubated with rabbit anti-SF polyclonal antibody (harvested in Section 2.3) at a dilution of 1:50 in a humidified chamber at 37 °C for 60 min. After three washes with PBST (PBS with 0.1% Tween-20), the cells were incubated for 50 min at 37 °C with the Horseradish peroxidase (HRP)-labeled goat anti-rabbit IgG (Beyotime Biotech Inc., Shanghai, China) at a dilution of 1:200 in PBST. The cells were again washed 3 times with PBST, followed by incubation for 4–5 min at room temperature in diaminobenzidine solution (Solarbio, Beijing, China). Cell staining was examined using Nikon light microscope (Nikon corporation, Tokyo, Japan).

## 3. Results

### 3.1. Prediction of BCEs on S^CV777^ and S^SD2014^

**Figure 1:**
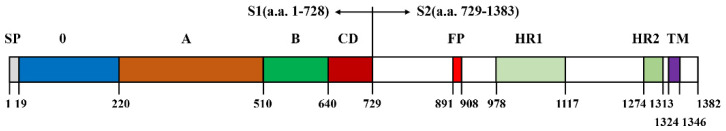
Schematic diagram of porcine epidemic diarrhea virus (PEDV) spike (S) protein. Different domains of S protein of PEDV CV777 (S^CV777^) (ALS35469.1) are colored. SP, signal peptide; 0, S1^0^; A, S1^A^; B, S1^B^; CD, S1^CD^; FP, fusion peptide; HR1, heptapeptide repeat 1; HR2, heptapeptide repeat 2; and TM, transmembrane domain. Numbers shown below the long bar denote the beginning and end amino acids of each domain.

**Figure 2:**
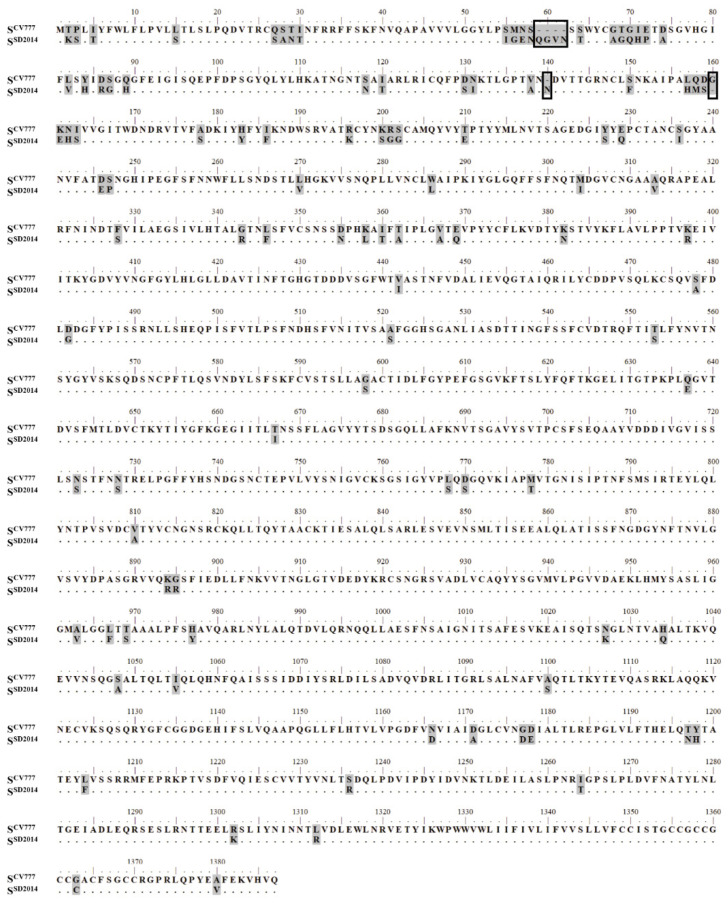
Amino acid sequence comparison of S^CV777^ and S protein of PEDV SD2014 (S^SD2014^). Amino acid sequences of S^CV777^ and S^SD2014^ (AND76936) were aligned with Mega 7. Differential amino acids were shaded with grey. The identical amino acid in the sequence of S^SD2014^ was substituted with a “.”; The amino acid in rectangle was the insertion/deletion site.

As in other alphacoronaviruses, the PEDV S protein can be divided into S1 and S2 subunits, and each subunit is composed of several functional domains (Figure 1). The aa sequence identity of S^CV777^ and S^SD2014^ was 92.21%. Compared to S^CV777^, S^SD2014^ has 108 differential residues including the insertion of ^59^QGVN^62^ and ^140^N and the deletion of ^160^G typically found in the S protein of variant PEDV strains. Most of the differential residues in the S1 subunit are in the S1^0^ domain (46/80) and S1^A^ domain (23/80), while the 28 differential residues in the S2 subunit scatter over different functional domains except fusion peptide (FP) and transmembrane domain (TM) (Figure 2).

Thirty-five and 34 BCEs were predicted on S^CV777^ and S^SD2014^, respectively (Table 1). Among the predicted epitopes, 15 BCEs on S^CV777^ and 19 BCEs on S^SD2014^ have an antigenicity value greater than 0.4, which is the default threshold for viral antigen by Vaxijen 2.0, and were coined as dominant BCEs. E7 was predicted to have the highest antigenicity at position 186 to 192 aa of S^CV777^ (2.1198), while the strongest antigenicity on S^SD2014^ was predicted at position 133 to 146 aa with an antigenic value of 1.0705 (Table 1). No epitope was predicted on the signal peptide (SP), FP, and TM domains of the two S proteins.

Compared to S^CV777^, amino acid variations on S^SD2014^ led to the generation of five new BCEs located on the S1^0^ (E5), S1^B^ (E14 and E17), ahead of FP (E21), and heptapeptide repeat 1 (HR1) (E27) domains, respectively, and elimination of six previous BCEs, which were distributed on the S1^0^ (E5 and E8), S1^A^ (E14 and E17), S1^CD^ (E21), and HR1 (E29) domains. Similarly, amino acid variation on S^SD2014^ generated nine new dominant epitopes, distributed on the S1^0^ (E3, E5, and E6), S1^A^ (E11), S1^B^ (E14 and E17), ahead of FP (E20 and E21), and HR1 (E27) domains and eliminated five previous dominant BCEs, which were distributed on the S1^A^ (E17), S1^CD^ (E22), and ahead of heptapeptide repeat 2 (HR2) (E30 to E32) domains.

### 3.2. Reactivity of Acquired Anti-S Sera with PEDV DR13

**Figure 3:**
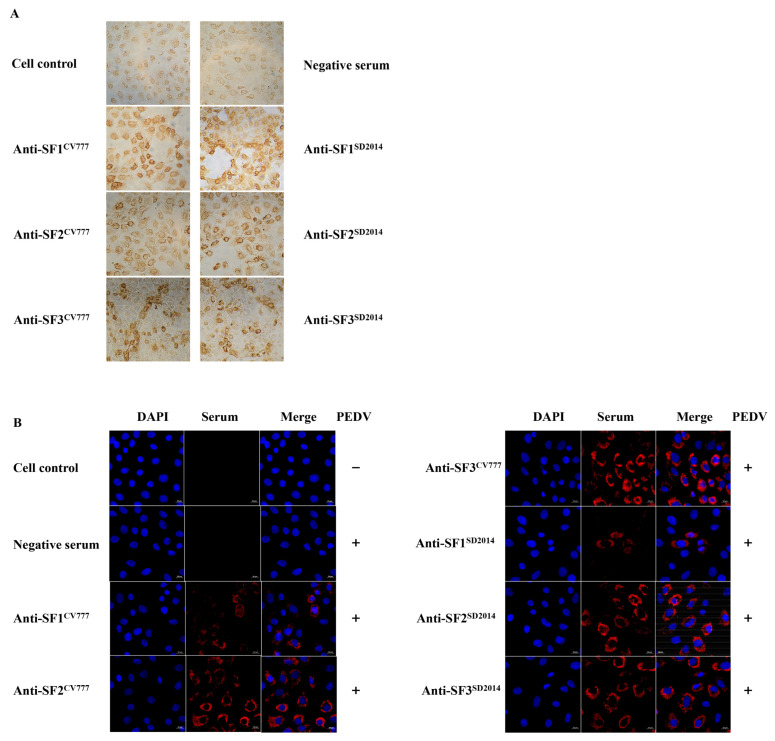
Reactivity of anti-S sera with attenuated PEDV DR13 detected by immunohistochemistry assay (IHC) (**A**) and immunofluorescence assay (IFA) (**B**). Cells were infected with PEDV DR13 at multiplicities of infection (MOI) of 0.1, and at 18 h postinfection (h.p.i.), cells were fixed and stained with anti-S sera. For IHC, Horseradish Peroxidase (HRP)-labeled goat anti-rabbit IgG was used as the secondary antibody. Then cells were incubated with diaminobenzidine before visualization with a light microscope. For IFA, Alexa Flur 647 goat anti-rabbit IgG (H + L) was used as the secondary antibody, and 4’,6-diamidino-2’-phenylindole (DAPI) was used to visualize the cell nuclei.

Before identifying linear BCEs on the S protein with the prepared rabbit anti-S sera, IFA and IHC were firstly performed to investigate whether the six anti-S segment sera could react with PEDV DR13. As shown in Figure 3A,B, all the six anti-S fragment sera could specifically recognize the PEDV DR13 strain. The negative control serum could not stain PEDV DR13 (Figure 3A,B). These results show that the acquired sera could bind to PEDV specifically. Although the results of IHC show that there is no obvious difference among the three anti-SF (SF1, SF2, and SF3) sera in the reactivity with the attenuated DR13 strain (Figure 3A), the IFA intensities with the anti-SF2 serum and anti-SF3 serum as the primary antibodies were obviously stronger than that with anti-SF1 serum as the primary antibodies (Figure 3B).

### 3.3. Reactivity of Anti-S Sera with S^CV777^ and S^SD2014^

**Figure 4:**
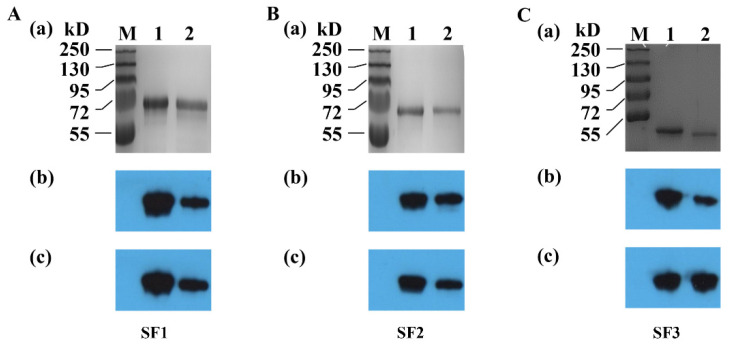
Reactivity of anti-S protein fragment 1 (SF1) sera with SF1 (**A**), anti-SF2 serum with SF2 (**B**), and anti-SF3 serum with SF3 (**C**). (**a**). SDS-PAGE analysis of purified SFs. (**b**,**c**). Western blot analysis with anti-SFs of PEDV CV777 (SFs^CV777^) and anti-SFs of PEDV SD2014 (SFs^SD2014^) sera as the primary antibody, respectively. Lane M: Protein marker; lane 1 and 2: Purified SFs^CV777^ and SFs^SD2014^, respectively.

To test the binding ability of the prepared rabbit sera with S^CV777^ and S^SD2014^, the purified S protein segments were separated through SDS-PAGE and transferred onto PVDF film for WB with the sera, respectively. As shown in Figure 4, the sera could bind to the corresponding protein segments from both strains, indicating that co-recognized epitopes exist in all the three fragments of the PEDV S protein.

### 3.4. Identification of Positive 16-Mers on S^CV777^

**Figure 5:**
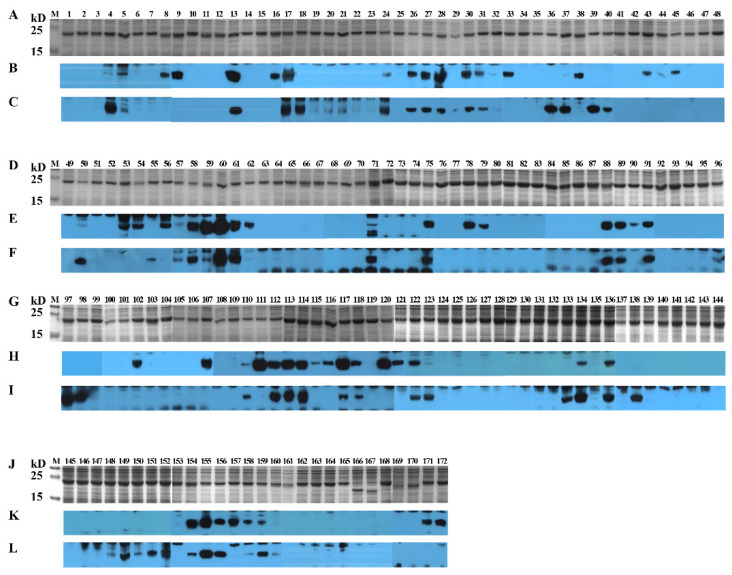
SDS-PAGE and Western blotting analysis of 16-mer- and 14-mer S^CV777^-derived peptides. One hundred and seventy-one 16-mer peptides and one 14-mer peptide covering the entire S^CV777^ were glutathione S-transferase (the initial 188 aa, GST)-fusion expressed in *Escherichia coli* (*E. coli*) BL21. After induction, cells were harvested by centrifugation, and cell pellets were resolved by SAS-PAGE using 15% gels. Gels were either stained or processed for Western blots. Polyvinylidene difluoride (PVDF) membranes were first blocked with 5% (*w*/*v*) skimmed milk, incubated sequentially with anti-S serum and goat anti-rabbit IgG, and then visualized by enhance chemiluminescence. (**A**,**D**,**G**,**J**): SDS-PAGE analysis of 171 GST-fusion expressed 16-mers and one 14-mer. (**B**,**E**,**H**,**K**): Western blot analysis of 171 GST-fusion expressed 16-mers and one 14-mer with anti-S^CV777^serum. (**C**,**F**,**I**,**L**): Western blot analysis of 171 GST-fusion expressed 16-mers and one 14-mer with anti-S^SD2014^ serum. 1–172: GST-fusion expressed 16-mers (P1–P171) and 14-mer (P172).

To identify linear BCEs on S^CV777^, serial 16-mers and a 14-mer overlapping 8 aa were GST-fusion expressed in *E. coli*. Among the 171 16-mers and the single 14-mer segments covering the entire S^CV777^, four 16-mers (P166, P167, P169, and P170) in TM domain and cytoplasmic tail (CT) could not be expressed. A little surprisingly, P168 (1337–1352 aa) at the junction of TM and CT was efficiently expressed. The GST-fusion 16-mers were expressed in size about 22.4 kD (Figure 5A,D,G,J).

In order to comprehensively study the BCE distribution on S^CV777^, anti-S^CV777^ serum and anti-S^SD2014^ serum were used to screen positive peptides from the serial GST-fusion expressed 16-mers (or 14-mers) by WB. Among 168 16/14-mers screened, 60 positive 16-mers and 1 positive 14-mer were identified by rabbit anti-S^CV777^ serum (Figure 5B,E,H,K), while 53 16-mers could be recognized by rabbit anti-S^SD2014^ serum (Figure 5C,F,I,L). In total, 78 positive reactive 16-mers and 1 14-mer were identified by WB using the two kinds of anti-sera, among which 35 16-mers (P4–P5, P13, P17, P24, P26–P28, P30–P31, P50, P57–P61, P71, P75, P88–P89, P91, P110, P112–P114, P117–P118, P122, P134, P136, P154–P156, and P158–P159) could be recognized by both anti-S^CV777^ serum and anti-S^SD2014^ serum and were coined as Co-RecoMers. Of the remaining positive 16-mers or 14-mer, 26 (P8–P9, P16, P32–P33, P38, P43–P45, P53–P54, P56, P62, P78–P79, P90, P102, P107, P111, P115–P116, P120–P121, P157, and P171–P172) and 18 (P18, P36–P37, P39–P40, P50, P55, P96–P98, P123, P133, P138, P148–P152, and P160) were specific for the anti-S^CV777^ serum and the anti-S^SD2014^ serum, respectively. Therefore, 77.2% of the 79 positive 16/14-mers of S^CV777^could be bound by its anti-serum, and 67.1% could be bound by anti-S^SD2014^ serum, indicating an obvious overlapping of antigenicity between the two S proteins.

**Figure 6:**
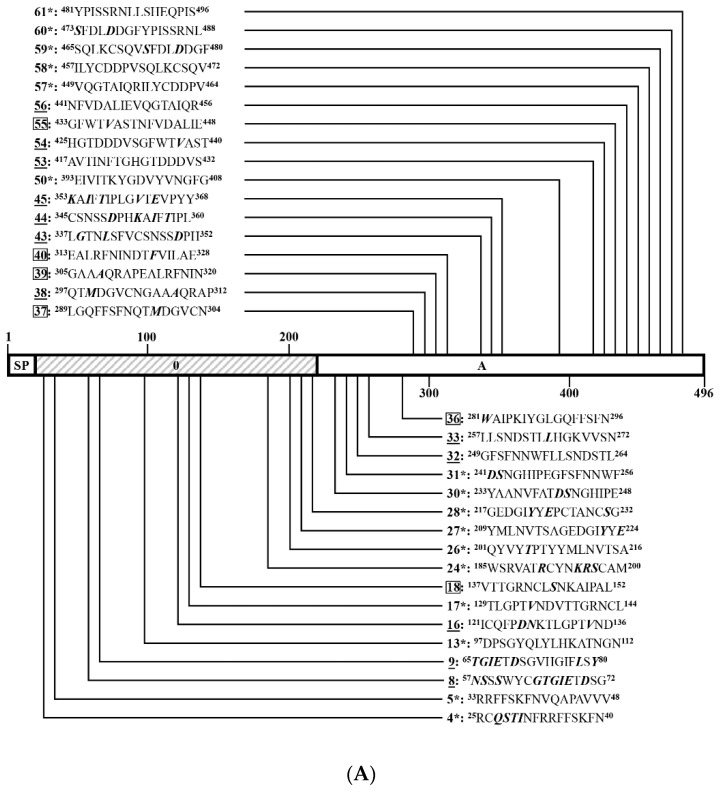

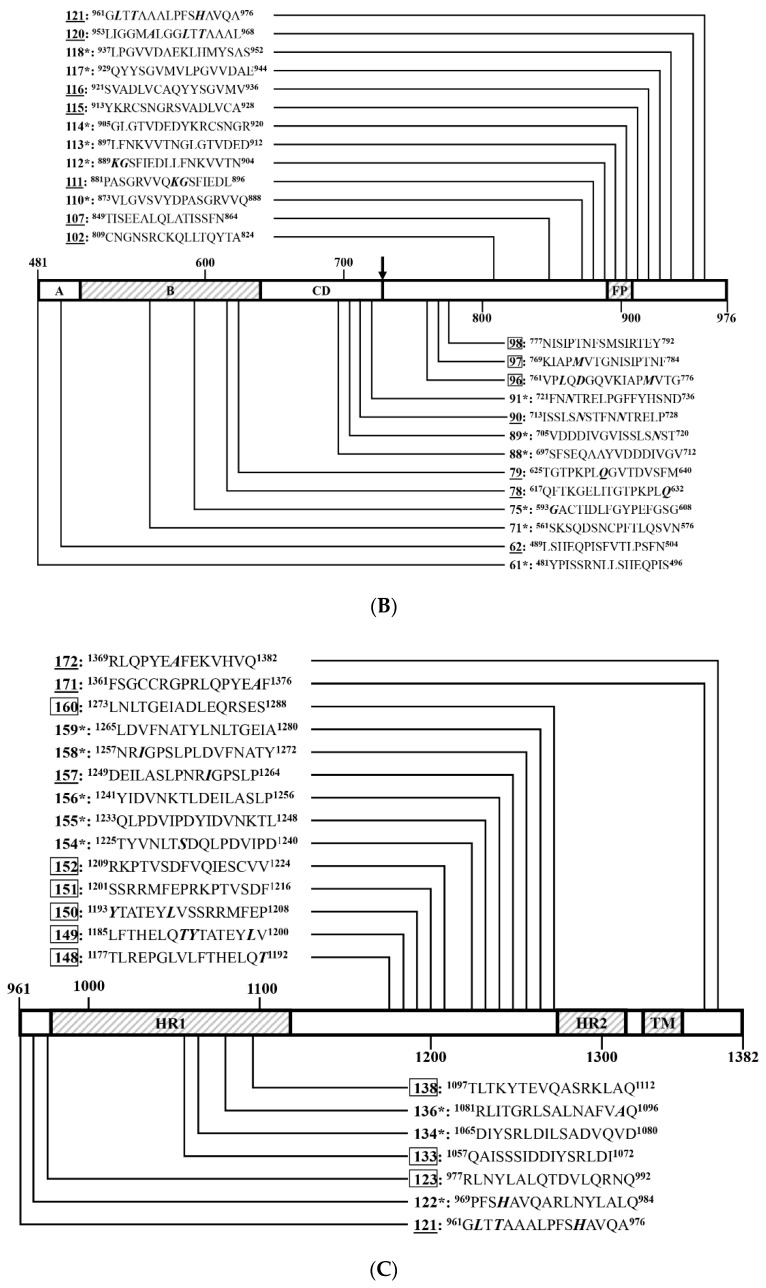
Distribution of positive peptides on SF1^CV777^ (**A**), SF2^CV777^ (**B**), and SF2^CV777^ (**C**). The horizontal bar represents the primary structure of S^CV777^ protein, of which the functional domains are framed as Figure 1A. Numbers assigned to the 16-mer or 14-mer peptides are the same as in Figure 5. Numbers underlined mark the peptides that are anti-S^CV777^ serum-positive; Numbers in boxes mark peptides that are anti-S^SD2014^ serum-positive; Numbers with “*” mark peptides recognized by both anti-S^CV777^ serum and anti-S^SD2014^ serum (Co-RecoMers). The positions of each peptide in the S^CV777^ protein were indicated with superscripts. The arrow showed the boundary of S1/S2.

Eleven (P4–P5, P8–P9, P13, P16–P18, P24, and P26–P27) out of the 79 positive 16/14-mers were located in the S1^0^ domain, including three anti-S^CV777^ serum-specific (P8–P9 and P16) and one anti-S^SD2014^ serum-specific (P18) (Figure 6A). Twenty-four positive 16-mers were in the S1^A^ domain, among which 10 (P32–P33, P38, P43–P45, P53–P54, P56, and P62) and 5 (P36–P37, P39–P40, and P55) were anti-S^CV777^ and anti-S^SD2014^ sera-specific, respectively. The other nine (P28, P30–P31, P50, and P57–P61) were Co-RecoMers (Figure 6A,B). Four positive 16-mers were in the S1^B^ domain; P71 and P75 were Co-RecoMers, and P78 and P79 were specific for the anti-S^CV777^serum (Figure 6B). Four positive 16-mers were in the S1^CD^ domain, among which P90 was anti-S^CV777^ serum-specific, and the other three (P88–P89 and P91) were Co-Recomers (Figure 6B). Six positive 16-mers were located in the region ahead of the FP domain of the S2 subunit, which included two anti-S^CV777^ serum-specific (P102 and P107) and three anti-S^SD2014^-specific (P96–P98) 16-mers and one Co-RecoMer (P110) (Figure 6B). Four positive 16-mers were identified on the FP domain, among which P111 was anti-S^CV777^ serum-specific, and P112–P114 were Co-RecoMers (Figure 6B). Six positive 16-mers were identified between FP and HR1, of which four (P115–P116 and P120–P121) were anti-S^CV777^serum-specific; the other two (P117–P118) were Co-RecoMers (Figure 6B,C). Six positive 16-mers were identified on the HR1 domain, of which three (P123, P133, and P138) were anti-S^SD2014^ serum-specific, and the other three (P122, P134, and P136) were Co-RecoMers (Figure 6C). Ten 16-mers were located ahead of HR2, of which one (P157) and five (P148–P152) were specific for the anti-S^CV777^ and anti-S^SD2014^ sera, respectively, and the remaining four (P154–P156 and P158) were Co-RecoMers (Figure 6C). Two 16-mers, one Co-RecoMers (P159) and one specific for the anti-S^SD2014^ serum (P160), were identified in HR2 domain (Figure 6C). One 16-mer (P171) and one 14-mer (P172) were in CT, both of which were specifically recognized by the anti-S^CV777^ serum (Figure 6C). No positive 16-mer was identified in the SP and TM domains as expected.

### 3.5. Identification of Positive 16-Mers on S^SD2014^

**Figure 7:**
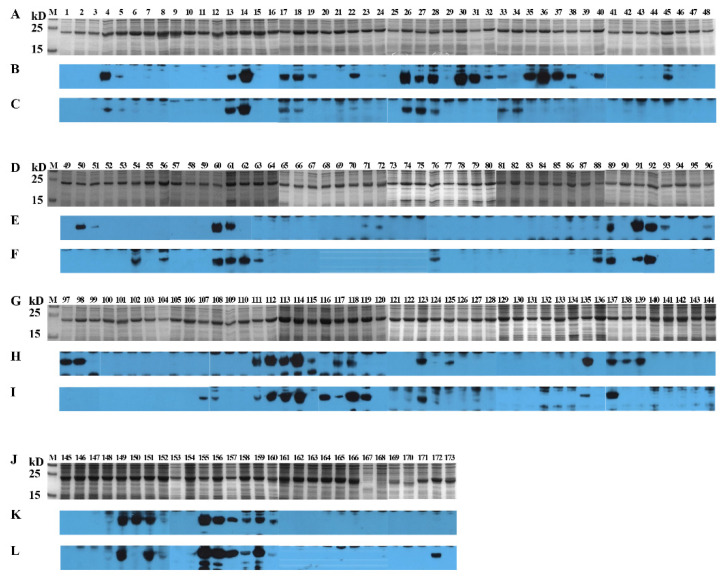
SDS-PAGE and Western blotting analysis of 16-mer- and 10-mer S^SD2014^-derived peptides. One hundred and seventy-two 16-mer peptides and one 10-mer peptide covering the entire S^SD2014^ were GST-fusion expressed in *E. coli* BL21. After induction, cells were harvested by centrifugation, and cell pellets were resolved by SAS-PAGE using 15% gels. Gels were either stained or processed for Western blots. PVDF membranes were first blocked with 5% (*w*/*v*) skimmed milk, incubated sequentially with anti-S serum and goat anti-rabbit IgG, and then visualized by enhance chemiluminescence. (**A**,**D**,**G**,**J**): SDS-PAGE analysis of 172 GST-fusion expressed 16-mers and one 10-mer. (**B**,**E**,**H**,**K**): Western blot analysis of 172 GST-fusion expressed 16-mers and one 10-mer with anti-S^SD2014^ serum. (**C**,**F**,**I**,**L**): Western blot analysis of 172 GST-fusion expressed 16-mers and one 10-mer with anti-S^CV777^ serum. 1–173: GST-fusion expressed 16-mers (P1–P172) and 10-mer (P173).

Using the same strategy as in 3.4 to identify linear BCEs on S^CD2014^, both anti-S^CV777^ and anti-S^SD2014^ sera were used to screen the positive peptides fusion-expressed with GST tags. Except for the 4 16-mers (P167–P170), which span part of the TM domain and its immediate downstream, the other 168 GST-fusion 16-mers or one 10-mer were successfully expressed (Figure 7). From the 169 expressed peptides, 56 and 45 positive 16-mers were identified by anti-S^SD2014^ and anti-S^CV777^ sera, respectively (Figure 7). Among the positive 16-mers identified, 33 (P4–P5, P13–P14, P17–P18, P22, P26–P28, P33, P60–P61, P89, P91–P92, P111–P114, P117–P118, P123, P135, P137, P149, P151, and P155–P160) were Co-RecoMers (Figure 7). Twelve positive 16-mers (P34, P54, P56, P62–P63, P76, P88, P107–P108, P116, P119, and P172) were anti-S^CV777^serum-specific, and 23 16-mers (P19, P30–P32, P35–P38, P40, P45, P50–P51, P71–P72, P93, P96–P98, P115, P125, P138–P139, and P150) were anti-S^SD2014^ serum-specific. Therefore, 68 positive 16-mer were totally identified on S^SD2014^, of which 66.2% could be recognized by anti-S^CV777^ serum, and 82.4% could be bound by their own anti-serum.

**Figure 8:**
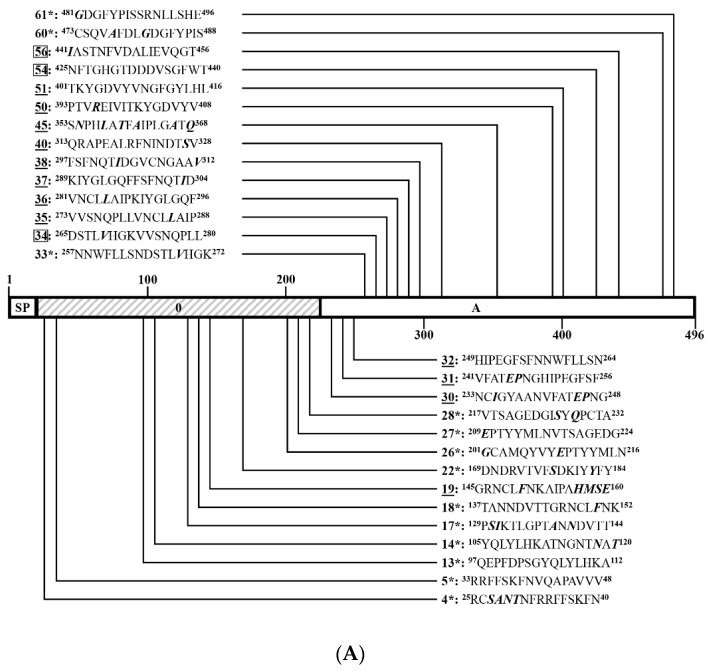

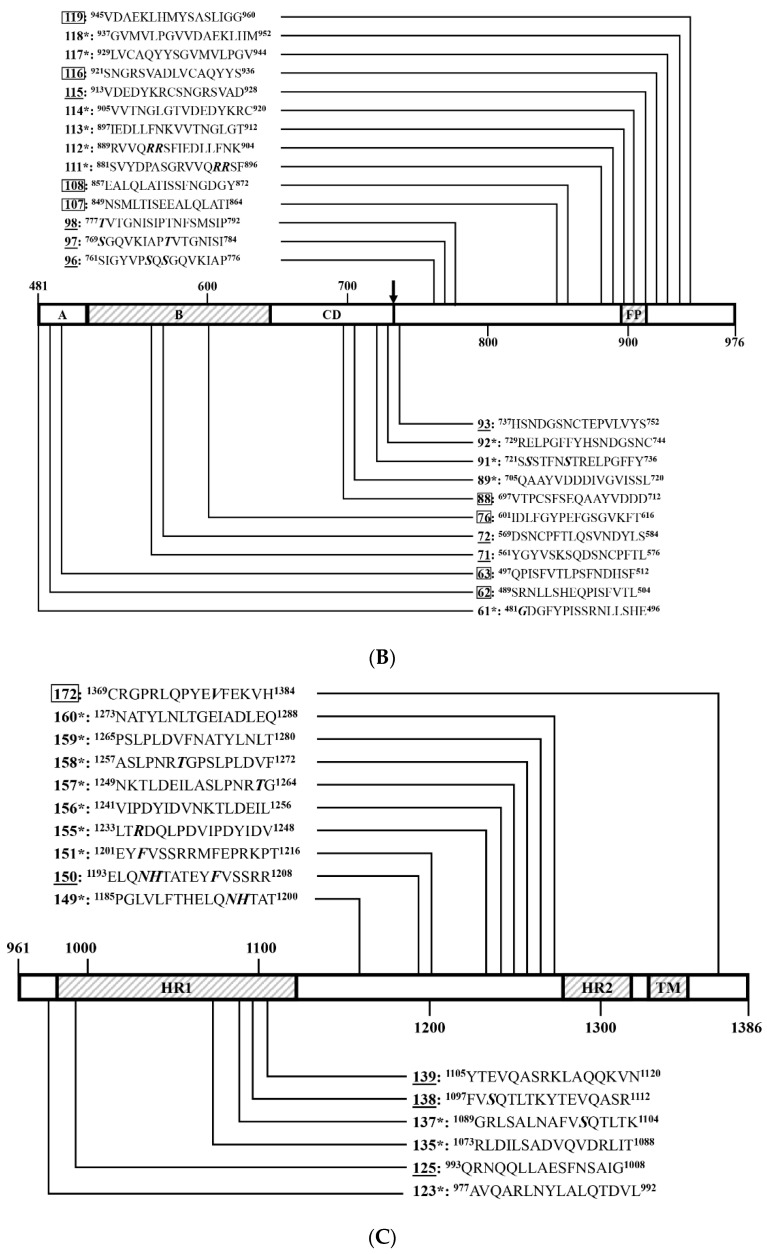
Distribution of positive peptides on SF1^SD2014^ (**A**), SF2^SD2014^ (**B**), and SF2^SD2014^ (**C**). The horizontal bar represents the primary structure of S^SD2014^ protein, of which the domains were framed as in Figure 1. Numbers assigned to the 16-mer or 10-mer peptides are the same in Figure 7. The numbers underlined mark peptides that are anti-S^SD2014^ serum-positive. The numbers in boxes mark the peptides that are anti-S^CV777^ serum-positive; Numbers with “*” mark peptides recognized by both anti-S^CV777^ serum and anti-S^SD2014^ serum (Co-RecoMers). The positions of each peptide in S^SD2014^ protein are indicated with superscripts. The arrow shows the boundary of S1/S2.

Like the distribution of positive 16-mers on S^CV777^, no positive 16-mer was located at the SP and TM domain of S^SD2014^. Ten positive 16-mers (P4–P5, P13–P14, P17–P19, P22, and P26–P27) were in the S1^0^ domain, among which P19 is anti-S^SD2014^-specific, and the other nine were Co-RecoMers (Figure 8A) (9/10). This finding was very special, as there was a high rate (90%) of Co-Recomers in this area. Twenty positive 16-mers were in the S1^A^ domain, including 11 anti-S^SD2014^ serum-specific (P30–P32, P35–P38, P40, P45, and P50–P51), 5 (P34, P54, P56, and P62–P63) anti-S^CV777^ serum-specific, and 4 Co-RecoMers (P28, P33, and P60–P61) (Figure 8A,B). That means in the S1^A^ segment, the anti-S^CV777^ serum-specific 16-mers take 42% of the positive 16-mers (9/21). Two (P71–P72) and one (P76) positive 16-mers in the S1^B^ domain were specific for anti-S^SD2014^ and anti-S^CV777^ sera, respectively (Figure 8B) (1/3). One (P88) of the four positive 16-mers in the S1^CD^ domain was anti-S^CV777^ serum-specific, and the other three (P89 and P91–P92) were Co-RecoMers (Figure 8B) (4/4). Eight positive 16-mers were in the region ahead of FP, among which four (P93 and P96–P98) and two (P107–P108) were anti-S^SD2014^ and anti-S^CV777^ sera-specific, respectively, and the other two (P111–P112) were Co-RecoMers (Figure 8B) (4/8). The two positive 16-mers (P113–P114) in FP were Co-RecoMers (Figure 8B) (2/2). Five positive 16-mers were located ahead of HR1, of which P115 was specific for anti-S^SD2014^ serum, and P116 and P119 were specific for anti-S^CV777^ serum, and the other two (P117–P118) were Co-RecoMers (Figure 8B) (4/5). Six positive 16-mers were in the HR1 domain, of which three (P125 and P138–P139) were specific for anti-S^SD2014^ serum, and the other three (P123, P135, and P137) were Co-RecoMers (Figure 8C) (3/6). Eight positive 16-mers located ahead of HR2, including 7 Co-RecoMers (P149, P151, and P155–P159) and one anti-S^SD2014^ serum-specific (P150) (Figure 8C) (7/8). One Co-RecoMer (P160) was identified in HR2 domain (Figure 8C) (1/1). In the CT of S^SD2014^, only one anti-S^CV777^ serum-specific 14-mer (P172) was identified (Figure 8C) (1/1).

### 3.6. An Overview of Positive 16-Mers on S^CV777^ and S^SD2014^

**Figure 9:**
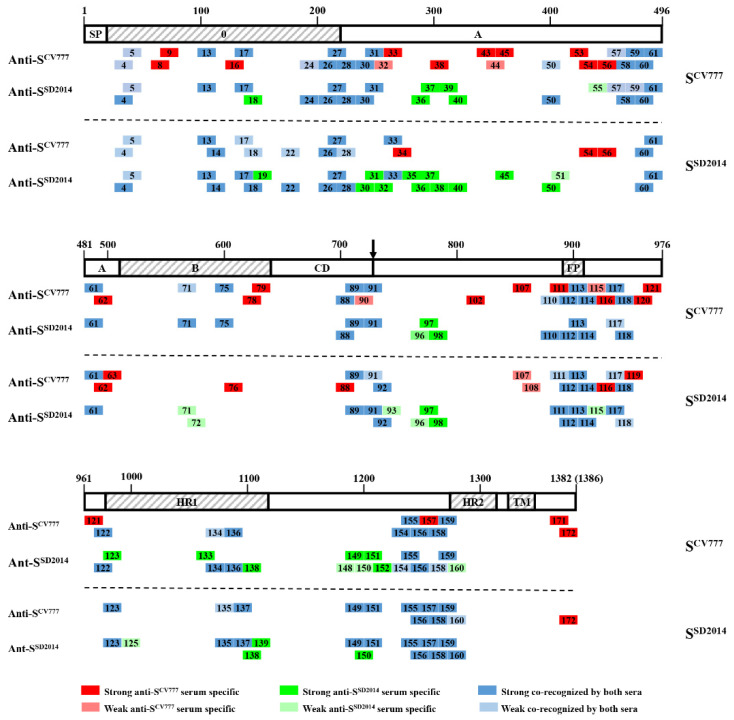
Comparative overview of positive 16-mers on S^CV777^ and S^SD2014^. The horizontal bar represents the primary structure of the S protein, of which the functional domains are framed as Figure 1. Numbers shown above the long bar denote the beginning and end amino acids of the S protein of PEDV. The short rectangles are positive 16-mers of S^CV777^ or S^SD2014^ (except P172, which was a 14-mer on S^CV777^). Short rectangles filled with red color are anti- S^CV777^ serum-specific 16-mers. Short rectangles filled with green color are anti-S^SD201^ serum-specific 16-mers. Short rectangles filled with blue color are Co-RecoMers. Numbers assigned to the 16-mer peptides are the same as in Figure 5 or Figure 7. The arrow show the boundary of S1/S2.

Among the 68 Co-Recomers found in the two S proteins, only 20 (P4–P5, P13, P17, P26–P28, P60–P61, P89, P91, P112–P114, P117–P118, P155–P156, and P158–P159) were Co-Recomers on both S proteins (coined as Shared Co-RecoMers here) (Figure 5, Figure 7 and Figure 9). Nine of the Shared Co-Recomers resided in the high variant domains, S1^0^ (5) and S1^A^ (4).

Some Co-RecoMers in one S protein had their corresponding positive 16-mers in the other S protein, but they could not be bound by both sera anymore. Five Co-RecoMers on S^CV777^ were no longer Co-RecoMers on S^SD2014^. These 16-mers on S^SD2014^ could only be bound by anti-S^SD2014^ serum (P30–P31, P50, and P71) or anti-S^CV777^ serum (P88). Likewise, eight Co-RecoMers on S^SD2014^ were not Co-RecoMers on S^CV777^. These 16-mers on S^CV777^ could only be recognized by anti-S^CV777^ serum (P33, P111, and P157) or anti-S^SD2014^ serum (P18, P123, P149, P151, and P160) (Figure 9).

There were 18 positive 16-mers, which appeared parallel on both proteins, but they were not Co-RecoMers. They could only be bound by one serum: Four positive 16-mers (P32, P38, P45, and P115) on the two S proteins could only be bound by their own anti-sera. Six positive 16-mers (P54, P56, P62, P107, P116, and P172) on the two proteins could only be bound by anti-S^CV777^ serum. On the opposite, eight 16-mers (P36–P37, P40, P96–P98, P138, and P150) on the two proteins could only be bound by anti-S^SD2014^ serum (Figure 9). Therefore, there were different kinds of 16-mers that existed parallel on the two S proteins: 20 Shared Co-RecoMers, 13 one-sided Co-RecoMers, and 18 non-Co-Recomers.

Among the positive 16-mers that resided in either of the two proteins, 28 16-mers were identified only on S^CV777^, which included 10 Co-RecoMers (P24, P57–P59, P75, P110, P122, P134, P136, and P154), 13 anti-S^CV777^ serum-specific (P8, P9, P16, P43–P44, P53, P78–P79, P90, P102, P120–P121, and P172), and 5 anti-S^SD2014^ serum-specific (P39, P55, P133, P148, and P152) (Figure 9). Meanwhile, 16 positive 16-mers were identified only on S^SD2014^, including 5 Co-RecoMers (P14, P22, P92, P135, and P137), 7 anti-S^SD2014^ serum-specific (P19, P35, P51, P72, P93, P125, and P139), and 5 anti-S^CV777^-specific (P34, P63, P76, P108, and P119) (Figure 9).

Collectively, among the 68 BCEs identified on S^SD2014^, only 13 (19.1%) Shared Co-Recomers in the S1^0^ (5), S1^A^ (2), S1^CD^ (1), ahead of FP (1), FP (2), and ahead of HR2 (2) domains manifested the same antisera binding property as the corresponding Shared Co-Recomers on S^CV777^, which indicate that the incidence of exactly conserved BCE shared by the two S proteins was only 19.1%. The remaining 55 BCEs in the S1^0^ (5), S1^A^ (18), S1^B^ (3), S1^CD^ (3), ahead of FP (7), ahead of HR1 (5), HR1 (6), ahead of HR2 (6), HR2 (1), and CT (1) domains exhibited more or less difference in antisera binding ability compared with the corresponding 16-mers on S^CV777^, even with the other 7 Shared Co-Recomers. Furthermore, 28 BCEs on S^CV777^ (S1^0^, 4; S1^A^, 8; S1^B^, 3; S1^CD^, 1; ahead of FP, 2; ahead of HR1, 2; HR1, 4; ahead of HR2, 3; and CT, 1) were missing on S^SD2014^, while 17 new BCEs formed on S^SD2014^ (S1^0^, 3; S1^A^, 4; S1^B^, 2; S1^CD^, 1; ahead of FP, 2; ahead of HR1, 1; and HR1, 4). The above analysis demonstrates that the BCE conformity rate between the two strains was only 19.12% (13/68), albeit 66.2% of BCEs on S^SD2014^ could be recognized by anti-S^CV777^ serum. A total of 35.4% (28/79) of the BCEs distributed on nearly all functional domains (except FP and HR2) of S^CV777^ were obliterated from S^SD2014^, but at the same time, a quarter (17/68) of BCEs were newly formed on the latter

## 4. Discussion

Since late 2010, the highly virulent PEDV variants have led to heavy economic loss to the world swine industry due to the high mortality rate of infected newborn piglets [20,21]. The vaccination of sows with commercial vaccines based on classical strains, such as CV777, could not provide satisfactory protection in piglets [2,22]. Antigenic variations, especially variations in the S protein, between classical and emerging PEDV strains were proposed to be the reason for the failure of CV777 strain-based vaccines [8,22], but sufficient evidence for the theory was warranted. To address this issue, we compared the differences of linear BCEs on the S proteins of the CV777 vaccine strain (S^CV777^) and a virulent variant strain SD2014 (S^SD2014^) using a bioinformatics approach and experimental methods. We found that with a sequence similarity of 92.21% between S^CV777^ and S^SD2014^, only 66.2% polypeptides (16-mers) of S^SD2014^ could be recognized by the anti-S^CV777^ serum. The parallel existing Co-Recomers (shared Co-RecoMers) took percentages of 29.4 and 25.3 for S^SD2014^ and S^CV777^, respectively. The absolute BCE conformity rate between the two strains was only 19.12%. All these figures indicate a low conservation of antigenicity between the two S proteins and a considerate decrease in antigenicity if PEDV CV777 was the vaccine strain for wild-type PEDV prevention.

The prediction of B-cell epitopes by the in silico method has obvious advantages including faster outputs and lower costs. However, the process relies heavily on a combination of multiple methods based on different algorithms and experimental validation techniques [23,24,25,26]. A recent study also found that most of the predicted epitopes on the PEDV S protein could not be recognized by all the sera selected. This inconsistency was related not only to the prediction method itself but also to the background of the sera selected [26]. Although the BCEs were predicted with strict condition, positive 16-mers were identified only from 68.6% (24/35) of the predicted BCEs on S^CV777^ and 52.9% (18/34) of the predicted BCEs on S^SD2014^, and more than half of positive 16-mers were identified from non-predicted BCE locations of both proteins (43/79 on S^CV777^ and 36/68 on S^SD2014^). Even the 16-mers containing sequences of some of the top five antigenic epitopes (antigenicity > 0.8) predicted on S^CV777^ (P23, P24, P70, and P71) or on S^SD2014^ (P9, P96, and P158) were negative or weakly positive (Table 1 and Figure 5 and Figure 7). The results provide more evidence that bioinformation methods in predicting immunogenic and potentially protective epitopes were not reliable and needed experimental verification.

Although genetic variation leads to the formation of two genotypes of PEDV (GI and GII), and each genotype can be further divided into different subtypes, cross-protection between GI and GII was widely reported in previous studies [27,28,29,30]. For this reason, it is generally agreed that there is only one serotype of PEDV, and different genotypes share antigenic epitopes. While several cross-reactive monoclonal antibodies and their corresponding epitopes or binding regions were reported on the S protein [9,31,32,33,34], the positions of the shared epitopes on the S protein are poorly defined. In this study, we found that all sera prepared by immunizing rabbits with each of the three segments (SF1 to SF3) of S^CV777^ and S^SD2014^ could recognize the attenuated PEDV DR13 as analyzed by both IHC and IFA techniques (Figure 3). WB results also indicate that each anti-serum of the segments could recognize both the corresponding segments of S^CV777^ and the segments of S^SD2014^ as well (Figure 4). All these results demonstrate that shared epitopes exist in all three segments of the S protein. Positive 16-mers that could be bound by both anti-S^CV777^ and anti-S^SD2014^ sera were indeed identified on each of the three segments of the S protein in the study (Figure 9). In addition, the 20 shared Co-Recomers were distributed over all three segments of the S protein (Figure 9). A comparison of full-length PEDV spike sequences from field isolates showed that most sequence variation was found in the S1 subunit, but several cross-reactive (neutralizing) mAbs binding regions were also localized in the same area [9,31,32,34]. Eleven shared Co-Recomers overlapped with the binding regions of these mAbs suggesting that these shared 16-mers might be cross-reactivity epitopes recognized by these mAbs. In addition, nine new shared epitopes were identified in the S2 subunit, which were located within the FP (P112–P114), in the upstream region of the HR1 (P117–P118), and in the upstream region of the HR2 (P155–P156 and P157–P158), respectively. As the coronavirus S2 subunit plays important roles in the membrane fusion process [26], antibodies binding to these shared epitopes may result in inhibition of membrane fusion processes during viral cell entry of both genotype PEDVs. The identification of cross-reactive epitopes on the S protein not only provides epitope evidence for the serological cross-reaction and mutual neutralization between the GI and GII PEDV strains found in clinical trials, but also provides candidates for the development of a multi-epitope universal vaccine as well as for antibody detection.

The PEDV S protein contains different domains, whose roles in virus infection have been elucidated to varying degrees. The S1^0^ domain was shown to bind sialic acid to facilitate virus infection [9,35]. While the S1^A^ domain was speculated to interact with cell surface heparan sulfate as an attachment factor based on its structural similarity with the other *alphacoronavirus* S1^A^ domain, the S1^B^ domain was known as a receptor binding domain [9,26,36,37]. The S2 subunit, consisting of FP and two heptad repeats (HR1 and HR2) mediate virus-virus and virus–cell membrane fusion [27,36]. Antibodies binding to any of the functional domains of the S protein can interfere with the infection process of the viruses; therefore, potential antigenic epitopes contained in these domains have been drawing the attention of PEDV researchers. Neutralizing antibodies recognizing different domains of S1 or the region spanning the S1–S2 border of the PEDV S protein were reported [7,10,11,12,13]. Several linear epitopes were also identified in domains of the S1 subunit [11,12,31,33,38,39]. However, information of BCEs in the S2 subunit is limited, with only one neutralizing epitope identified [11,13,14]. In the present study, epitopes were predicted and identified by IEDB and WB, respectively, distributing in nearly all the functional domains of the S proteins of CV777 and SD2014, but the contour and composition of the epitopes on these two S proteins varied considerably (Table 1 and Figure 6, Figure 8 and Figure 9). On S^CV777^, 13 (16.5%) of the 79 BCEs were not bound by anti-S^SD2014^ serum, and 28 (35.4%) BCEs did not have counterparts on S^SD2014^. On the side of S^SD2014^, 16 (23.5%) of the 68 BCEs cannot be bound by anti-S^CV777^ serum, and 17 (25%) BCEs were newly formed compared with S^CV777^. The missed or non-bound BCEs scattered in different functional domains of the PEDV S protein except HR2. Especially in the receptor binding domain S1^B^ where the core neutralizing epitope is located, there was no shared Co-RecoMer in this domain. Furthermore, three out of the four BCEs of S^CV777^ were missing on S^SD2014^. Likewise, two out of the three BCEs in the S1^B^ domain of S^SD2014^ do not exist on S^CV777^. It is well known that change or deletion of antigenic epitopes in the neutralizing epitope region might lead to the immune escape of the variant strain against the classical strain-based vaccine [27,28,29]. The differential epitopes in multiple functional domains, especially in the neutralizing epitope-containing domains of the two PEDV S proteins, mean that the variant PEDV strain can escape the monitoring of antibodies in multiple steps of virus infection, such as receptor binding, S1/S2 cleavage, or membrane fusion process, to achieve the final immune escape.

BCEs are generally divided into linear BCEs and conformational BCEs [25]. Linear BCEs are continuous aa motifs in a protein, while conformational BCEs are aa clusters that are discontinuous in the primary structure but brought adjacent upon protein folding [40]. Although most neutralizing epitopes are conformational BCEs, and conformational BCEs require correct conformation to interact with their target antibody, short linear peptides (which can also be called linear BCEs) within the long conformational BCEs can be recognized by antibodies, and binding of these short linear BCEs to the antibody is sufficient to interfere with the neutralizing activity of the antibody [26]. On the other hand, neutralizing linear BCEs have also been identified on the S protein of PEDV [11,14,26]. In addition, accumulating evidence proves that cooperation among non-neutralizing antibodies can neutralize the virus, and non-neutralizing antibodies can strengthen the neutralization by a neutralizing antibody [33]. A previous study demonstrated that a non-neutralizing epitope peptide could significantly inhibit adsorption of PEDV to Vero cell surfaces [39]. Non-neutralizing antibodies have also been shown to play important roles in antiviral responses via antibody-dependent cellular cytotoxicity or Fc gamma receptor- and complement-dependent effector mechanisms in vivo [31]. The linear BCEs identified in this study will be the focus of future studies that evaluate their relevance to host antiviral responses.

In this study, rabbits instead of pigs were selected to generate antibodies because clean rabbits are easier to obtain than clean pigs, and rabbits were suitable for immune experiments to prepare a certain amount of serum based on economic consideration. As the natural host of PEDV, the sera from pigs immunized with PEDV S protein or more ideally, convalescent pigs infected with PEDV are theoretically preferred for BCE identification on the S protein. Future experiments will focus on identifying BCEs of the S protein by using high-quality porcine serum and check the possible bias from the findings using rabbit-generated antibodies.

## 5. Conclusions

Seventy-nine and sixty-eight linear BCEs (positive 16/14-mers) were identified on S^CV777^ and S^SD2014^, respectively, among which 20 BCEs were shared Co-Recomers. More than 40% of the BCEs recognized by anti-S^CV777^ serum on S^CV777^ cannot be recognized by anti-S^SD2014^ serum and vice versa. Therefore, compared with that of the classical vaccine strain CV777, antigenicity and immunity of S protein of variant PEDV strain SD2014 had changed significantly. Epitopes identified in this study provided a solid foundation for the rational design of multi-epitope-based vaccines and diagnostic techniques.

## Figures and Tables

**Table 1:** Predicted epitopes on S protein of PEDV CV777 (S^CV777^) and that of PEDV SD2014 (S^SD2014^).

S^CV777^				S^SD2014^			
No.	Position	Sequences	Antigenicity ^a^	No.	Position	Sequences	Antigenicity
E1	19–30	LPQDVTRCQSTIN	0.0361	E1	21–28	QDVTRCSA	−0.0665
E2	52–62	AVVVLGGYLPSMNSSSWY	0.1657	E2	55–64	IGENQGVNST	0.3703
E3	65–72	TGIETDSG	0.3072	***E3***	***70–76***	***QHPTASG***	***0.9738***
***E4***	***91–100***	***ISQEPFDPSG***	***0.6991***	***E4***	***95–105***	***ISQEPFDPSGY***	***0.6324***
E5	102–110	QLYLHKATN	−0.3804				
				***E5***	***111–120***	***KATNGNTNAT***	***0.4115***
E6	125–142	QLYLHKATNGNTSAIARLRICQFPDNKTLGPTVNDVTTGRN	0.3121	***E6***	***133–146***	***TLGPTANNDVTTGR***	***1.0705***
***E7***	***186–192***	***SRVATRC***	***2.1198***	***E7***	***188–194***	***DWSRVAT***	***0.5834***
E8	195–202	KRSCAMQY	0.2537				
E9	215–223	SAGEDGIYY	−0.0055	E8	219–233	SAGEDGISYQPCTAN	0.0702
E10	226–232	CTANCSG	−0.4497				
E11	238–248	FATDSNGHIPE	−0.7913	E9	244–253	TEPNGHIPEG	−0.7045
***E12***	***297–310***	***QTMDGVCNGAAAQR***	***0.4745***	***E10***	***301–307***	***QTIDGVC***	***1.0282***
E13	347–353	NSSDPHK	0.2269	***E11***	***350–356***	***SNSSNPH***	***0.4187***
E14	372–378	KVDTYKS	−1.422				
***E15***	***423–432***	***TGHGTDDDVS***	***0.5537***	***E12***	***427–436***	***TGHGTDDDVS***	***0.5537***
***E16***	***461–475***	***DDPVSQLKCSQVSFD***	***0.8676***	***E13***	***465–473***	***DDPVSQLKC***	***0.4517***
***E17***	***483–490***	***ISSRNLLS***	***0.4671***				
				***E14***	***520–526***	***SFGGHSG***	***0.4737***
E18	528–535	SDTTINGF	−0.3571	E15	530–539	IASDTTINGF	−0.4022
***E19***	***555–573***	***NSYGYVSKSQDSNCPFTLQ***	***0.8752***	***E16***	***559–575***	***NSYGYVSKSQDSNCPFT***	***0.7085***
				***E17***	***608–613***	***EFGSGV***	***0.6906***
E20	622–634	ELITGTPKPLQGV	0.1569	E18	627–640	LITGTPKPLEGVTD	0.2387
E21	688–694	GAVYSVT	0.0153				
***E22***	***716–728***	***LSNSTFNNTRELP***	***0.4482***	E19	720–732	LSSSTFNSTRELP	0.2311
E23	734–744	SNDGSNCTEPV	0.3639	***E20***	*7**38–747***	***SNDGSNCTEP***	***0.4006***
				***E21***	***762–775***	***IGYVPSQSGQVKIA***	***0.8513***
***E24***	***800–817***	***VSVDCVTYVCNGNSRCKQ***	***0.8176***	***E22***	***803–819***	***PVSVDCATYVCNGNSRC***	***0.5376***
E25	860–869	ISSFNGDGYN	−0.77	E23	865–873	SSFNGDGYN	−0.7927
***E26***	***875–887***	***GVSVYDPASGRVV***	***0.5215***	***E24***	***879–893***	***GVSVYDPASGRVVQR***	***0.6336***
E27	904–921	NGLGTVDEDYKRCSNGRS	0.2071	E25	906–925	VTNGLGTVDEDYKRCSNGRS	0.2239
E28	1013–1024	VKEAISQTSNGL	0.0891	E26	1017–1030	VKEAISQTSKGLNT	0.3561
E29	1037–1043	VVNSQGS	−0.0001				
				***E27***	***1064–1070***	***SSSIDDI***	***0.4527***
***E30***	***1118–1131***	***CVKSQSQRYGFCGG***	***0.6518***	E28	1101–1135	TLTKYTEVQASRKLAQQKVNECVKSQSQRYGFCGG	0.2232
***E31***	***1205–1214***	***MFEPRKPTVS***	***0.4124***	E29	1194–1218	LQNHTATEYFVSSRRMFEPRKPTVS	0.0433
***E32***	***1230–1243***	***TSDQLPDVIPDYID***	***0.4996***	E30	1233–1246	LTRDQLPDVIPDYI	0.3167
***E33***	***1256–1262***	***PNRIGPS***	***1.1446***	***E31***	***1259–1267***	***LPNRTGPSL***	***0.9542***
***E34***	***1280–1297***	***ADLEQRSESLRNTTEELR***	***0.615***	***E32***	***1284–1300***	***ADLEQRSESLRNTTEEL***	***0.5571***
E35	1348–1373	TGCCGCCGCCGACFSGCCRGPRLQPY	−0.2869	E33	1351–1361	STGCCGCCGCC	−0.5985
				E34	1364–1376	CFSGCCRGPRLQP	0.1851

Note: ^a^, Epitopes with antigenicity that scored higher than 0.4 were coined as dominant B cell epitopes (BCEs) and are shown in bold italics.

## Data Availability

Not applicable.
